# The effects of herbal medicine on epilepsy

**DOI:** 10.18632/oncotarget.16801

**Published:** 2017-04-03

**Authors:** Wei Liu, Tongtong Ge, Zhenxiang Pan, Yashu Leng, Jiayin Lv, Bingjin Li

**Affiliations:** ^1^ Jilin Provincial Key Laboratory on Molecular and Chemical Genetic, The Second Hospital of Jilin University, Changchun 130041, PR China; ^2^ Third Hospital of Jilin University, Changchun 130033, PR China

**Keywords:** herbal medicine, epilepsy, GABA, neuron, anticonvulsants

## Abstract

Traditional herbal medicine plays a significant role in the treatment of epilepsy. Though herbal medicine is widely used in antiepileptic treatment, there is a lack of robust evidence for efficacy and toxicity of most herbs. Besides, the herbal medicine should be subject to evidence-based scrutiny. In this context, we present a review to introduce the effects of herbal medicine on epilepsy. However, hundreds of herbal medicines have been investigated in the available studies. Some commonly used herbal medicines for epilepsy have been listed in our study. The overwhelming majority of these data are based on animal experiments. The lack of clinical data places constraints on the clinical recommendation of herbal medicine. Our study may conduct further studies and provide some insight on the development of anti-epileptic drugs.

## INTRODUCTION

Herbal medicine for epilepsy therapy is a centuries-old practiced medical form in diversified cultures [1–7]. The herbal medical traditions are reported in China, Iran, Europe, America. Chinese herbal medicine, which is significantly different from others, is time-honored tradition and based on sophisticated medical theories under-going long-term repeated confirmation. Besides, the quantity of the herbs for epilepsy in Chinese traditional herbal medicine is absolutely dominant in herbalism worldwide. Herbal medications are currently the most usual approach to complementary and alternative medications, which play an important part in the therapy to control epileptic seizures or complications caused by antiepileptic drugs [8]. The aims and attitudes of the patients with epilepsy who have access to herbal medicine in developing countries are different from those in the developed countries. The herbal medications, the most usual form of complementary and alternative medications, in developed countries are served for seizure control, reducing complications caused by antiepileptic drugs and general health maintenance, and the majority of the patients who take herbal medications do not reveal information to the physicians. The herbal therapies in developing countries are often used as a substitute for orthodox Western scientific medicine and supplementary treatment [8].

Though herbal medicine is accepted worldwide and extensively used in antiepileptic treatment, there is a lack of robust evidence for efficacy and toxicity of most herbs. The theory of traditional Chinese herbal medicine is essentially different from Western medicine. In Chinese medicine theory, the human body is not only an integral whole consist of internal organs, “channel” and fundamental substances such as essence, vital energy, blood and body fluid, but an inseparable part of outside natural world. “Channel” network connects internal organs as a functional group. So, the theories of traditional Chinese medicine are based on a philosophical system summarized by ancient people. As opposed to traditional Chinese medicine, Western medicine explores the underlying mechanism of biological activity and pathophysiological processes. Traditional Chinese medicine is often unintelligible for Western-trained physicians and needs scientific explanations for mechanism of “channel” and active components of herbs [9]. Though herbal medicine for epilepsy is widely used, it should be subject to evidence-based scrutiny. Thousands of studies have been reported that herbal medicine is used for epilepsy, most of which are experimental tests in laboratory animals. However, there is still a lack of critical analysis on efficacy and adverse effects in clinical use of herbal medicine for epilepsy. It is reported that approximately 30% patients with epilepsy who take antiepileptic drugs still suffer from seizures [10]. In addition, the reasons why the patients with epilepsy take herbal medicine include economic factors, cultural attitudes toward orthodox Western scientific medicine, substitute for artificial medicine that fails to control seizure. Therefore, herbal medicine may provide high possibility for scientists to find new molecular mechanism of epilepsy and new antiepileptic drugs. In this context, we present a review to introduce the effects of herbal medicine on epilepsy.

### Traditional herbal medicine for epilepsy in regions

#### Traditional herbal medicines for epilepsy in Asia

#### Iranian traditional herbal medicine for epilepsy

Iranian traditional medicine has a long history of herbal therapies for ailments, including convulsive seizures treatment. There are many Iranian traditional medicine books recording herbal medicine for epilepsy. A recent study has reviewed the herbal medicine for epilepsy recorded in five famous Iranian traditional medicine books [11]. The study has listed twenty-five herbal medicine for epilepsy for these books, and detailed the information about anticonvulsant effects of eleven herbals: paeonia officinalis, bryonia alba, lavandula stoechas, ferula persica willd, ferula asafoetida, coriandrum sativum, caesalpinia bonducella roxb, ferula gummosa boiss, cuscuta epithymum murray, cedrus deodara loudon, and origanum majorana. The extracts of used parts from most of these eleven herbal medicines exhibit antiepileptic effects on seizure animal models, while neighter ferula asafoetida nor ferula gummosa boiss can prevent pentylenetetrazol-induced seizures [11, 12]. In the mentioned data, some molecular mechanisms have been researched, as follows: the anticonvulsant activity of ethanolic extract of the aerial parts of bryonia alba results from its moderate affinity to the benzodiazepine-site of the gamma amino butyric acid (GABA) receptor [11], the aqueous-methanolic extract of lavandula stoechas may act as a calcium channel blocker [13], and the petroleum ether extract of caesalpinia bonducella may block the chloride ion channel linker to GABA receptors [14]. Besides, methanol Ferula extracts may have cytotoxic activity [12]. However, the lack of data and detailed information of herbal clinical medication on patients with epilepsy remains limited for herbal therapies and need to be further investigated.

Lavandula officinalis is another common herb used for epilepsy in Iranian traditional medicine. Some studies have suggested that it exhibits anticonvulsant effects on both pentylenetetrazol-induced seizures animal models and pentylenetetrazol kindling seizures in animal models [15, 16]. Besides, it is reported that it can protect cerebellar granular cells from glutamate-induced neurotoxicity [18]. The possible mechanisms of its antiepileptic effect have been demonstrated in some studies: inhibition of glutamate release, enhancement of GABA receptors [15], blockade of calcium channel, and its antioxidant effect [17–19]. The results indicate that Lavandula officinalis has huge potential for research and development of antiepileptic drugs.

#### Zizyphus jujube

Zizyphus jujube is used as an anticonvulsant in traditional medicine in India. Many studies have proven the antiepileptic properties in animal models assay [20, 21]. The hydroalcoholic extract of fruits of Zizyphus jujuba can decrease pentylenetetrazol- and MES-induced seizures in rats. Besides, the hydroalcoholic extract strongly enhances the anticonvulsant effects of phenytoin and phenobarbitone without serum level alternation of both drugs in combination therapy [21]. The antiepileptic effect may be mediated by inhibition of glutamate-induced over excitation, reduction of synaptic release of glutamate or NMDA (N-methyl-D-aspartate), or reversion antioxidant-oxidant imbalance [20, 21]. Moreover, zizyphus jujube can improve seizure-associated cognitive impairment and oxidative stress in rats [20]. However, these mechanisms have not been well evaluated.

#### Taxus wallichiana Zucc (Himalayan Yew)

Taxus wallichiana Zucc (Himalayan Yew) is a common native herbal medicine used in the northern areas of Pakistan and Nepal for epilepsy, though there is a lack of sufficient evidence on the anticonvulsant activities [22]. The anticonvulsant effect of Taxus wallichiana Zucc is firstly reported in a scientifically designed study in 1997, and demonstrates in pentylenetetrazol-induced seizures models by a recent study [23]. The mechanisms of anticonvulsant effect remain unknown, but there is potential to control myoclonic and absence seizures still leaving room for further studies.

#### Ganoderma lucidum

Ganoderma lucidum is a popular traditional herbal medicine for ailments in traditional Chinese medicine. Some studies conducted by a research team have revealed that Ganoderma lucidum spore has antiepileptic properties in *in vivo* and *in vitro* studies [24–27]. Ganoderma lucidum spore inhibits the expression of NF-κB in the brain of rats with epilepsy and the expression of N-Cadherin in hippocampal neurons, while enhancing neurotrophin-4 expression in hippocampal neurons [24]. The results from a recent research suggest that the antiepileptic effects of Ganoderma lucidum spore may also result from inhibition of the Ca^2+^ accumulated in epileptic hippocampal neurons and subsequent stimulation of CaMK IIα expression [28]. Though there is no report in our knowledge on the information of clinical use of Ganoderma lucidum spore, these studies indicate the potential of Ganoderma lucidum spore in the treatment of patients with epilepsy.

#### Salvia miltiorrhiza (Danshen or Chinese red sage)

Salvia miltiorrhiza is a traditional herbal medicine used for treating epileptic seizures in traditional Chinese medicine. Acetonic crude extracts of salvia miltiorrhiza have significant anticonvulsant efficacy in larval zebrafish-pentylenetetrazol models [10]. The active components are hydrophobic tanshinones, such as miltirone, 15, 16- Dihydrotanshinone I, cryptotanshinone, and tanshinone IIA. Compared with the other tanshinones, tanshinone IIA also exhibits anti-seizure effects on pentylenetetrazol induced mouse models and no sedative effects. The anti-seizure properties of tanshinone result from an interaction tendency with pathways irrelevant to GABA and related agonists [10]. The recent studies on tanshinones make it possible to be a potential anti-seizure drug.

#### Uncaria rhynchophylla

Uncaria rhynchophylla is another common herbal medicine in traditional Chinese medicine used for epilepsy. The antiepileptic effects of uncaria rhynchophylla have been seen in kainic acid-induced epileptic seizures models [29–31]. There are several compounds found in Uncaria rhynchophylla, and the neuroprotective related compounds of those are rhynchophylline and isorhynchophylline [32, 33]. Studies indicated that the anticonvulsant effects on kainic acid-induced epileptic seizures may be medicated by the regulation of immune response and neurotrophin signaling pathway, and inhibition of gene expression: neuron survival brain-derived neurotrophin factor (BDNF) gene and inflammation gene IL-1β [34]. Better yet, this study details the investigations of signal pathway and the studies on targets through genomics and immunohistochemistry, and it can bring some illumination to the mechanisms of research on antiepileptic herbal medicine.

#### Compound herbal medicine

Anti-epilepsy capsules (Kang Xian capsules), a compound of Shi Chang Pu(Rhi- zoma Acori Tatarinowii), Dan Nan Xing (Arisaema cum Bile), Tian Ma (Rhizoma Gastrodiae), Tai Zi Shen (Radix Pseudostellariae), Fu Ling (Poria), Chen Pi (Pericar- pium Citri Reticulatae), Ban Xia (Rhizoma Pinelliae), Zhi Qiao (Fructus Aurantii) and Chen Xiang (Lignum Aquilariae Resinatum), are clinically used for children epilepsy [35]. The clinical study of anti-epilepsy capsules on childhood epilepsy cases showed 57.4% cases were markedly effective, 25.9% effective, 10.3% improved, 5% ineffective and 1% aggravated. The total effective ratio was up to 83.3%. Besides, the frequency and duration of epilepsy had significantly decreased after taking anti-epilepsy capsules.

However, there are discrepancies between the effects of capsules on different types of epilepsy: better effects on autonomic epilepsy, and epilepsy caused by wind, phlegm, and terror while poor on paroxysmal epilepsy and epilepsy resulted from blood stasis. The antiepileptic effects of capsules are mediated by prolonging the attenuation duration of NMDA receptor channels, altering the expression of NMDA receptor subunits, and reducing in the concentration of intraneuronal Ca^2+^ inside the hippocampal neurons [36].

### Traditional herbal medicines for epilepsy in Africa

#### Acanthus montanus, et al

Herbal medicine is a common approach for epilepsy in traditional African medicine. It is reported that at least 43 plants have been traditionally used for epilepsy and convulsions, and the well-known herbal medicines include Acanthus montanus (*Acanthaceae*), Alchornea laxiflora (*Euphorbiaceae*), Hyptis spicigera (*Lamiaceae*), Microglossa pyrifolia Kuntze (*Asteraceae*), Piliostigma reticulatum (*Ceasalpiniceae*), and Voacanga africana (*Apocynaceae*) [37]. However, there are few researches on antiepileptic effects of the six cited plants. A recent study has investigated the extracts of these plants in several epilepsy relevant animal models [37]. The results suggest that there are different effects shown in these extracts. All extracts of the six plants inhibit strychnine-induced seizures, and Hyptis spicigera is the most effective. Five plants except Alchornea laxiflora decrease pentylenetetrazol-induced convulsions, with Hyptis spicigera being the most effective as well. The extracts of Acanthus montanus, Microglossa pyrifolia, and Voacanga africana prolong the time to the onset of convulsions induced by isonicotinic hydrazide acid and inhibit MES (maximal electroshock)-induced convulsions [37]. Besides, both the first two plants also exhibit significant anticonvulsant effects in picrotoxin-treated mouse [37]. Pharmacological diversities of these plants are mediated through a variety of mechanisms. The anticonvulsant effects of Alchornea laxiflora, Microglossa pyrifolia, Piliostigma reticulatum, and Voacanga Africana may result from antagonistic action on NMDA receptors. The antagonism of isonicotinic hydrazide acid- and pentylenetetrazol-induced seizures shown in the plant extracts may be mediated by interaction with GABAergic neurotransmission [38]. The protective effects against MES-induced convulsions are probably due to prolonging the inactivation of sodium channels [39]. A study on screen of traditional herbal medicines for epilepsy binding GABA_A_-benzodiazepine receptor assay suggests that some plants have shown to be active in binding to the GABA_A_-benzodiazepine receptor [40]. Though these results have made progress in Africa herbal medicine on epilepsy, the sophisticated analyses need to be further strengthened.

#### Ficus platyphylla

Ficus platyphylla is used in Nigeria's traditional medicine for epilepsy, especially northern Nigeria [41–44]. The efficacies of the plant are widely accepted in Nigeria's folk and the oral administration of the methanol extract of Ficus platyphylla stem bark is proven to have anticonvulsant effects and be safe in rats [44]. A recent scientific study has tested the extract in several animal seizures models (pentylenetetrazole-, strychnine-, picrotoxin-, isonicotinic hydrazide acid-, aminophylline- and MES- induced seizures) [41]. The psychoactive agents of the extract include saponins, flavonoids and tannins. The extract exhibits anti-seizure effects in pentylenetetrazol- and strychnine-induced seizures models, but fails to treat against sonicotinic hydrazide acid-, aminophylline-, and MES-induced seizures. Meanwhile, the extracts significantly delayed the latencies of myoclonic jerks and all models of seizures except MES-induced seizures. Besides, the extract can improve the learning impairment and ameliorate the neuronal cell loss in the cornu ammonis 1 and cornu ammonis 3 regions of hippocampus [42]. Possible mechanisms of antiepileptic activity mediated by the extract may attribute to inhibit of T-type Ca2+ currents, block glutamatergic neurotransmission mediated by the NMDA receptor, interfere with postsynaptic inhibition mediated by glycine, an important inhibitory transmitter to motor neurons and interneurons in the spinal cord, scavenge action of free radicals (such as reactive oxygen and nitrogen species), affinity for GABAergic and glutamatergic receptors, and reduction of glutamate release [41, 42]. The toxicity tests in rats showed that there were no toxic effects of the extract on rats, so it may be a potential drug for patients with epilepsy [41–44]. Nevertheless, there is no literature to show its efficiency in clinical trial.

#### Gladiolus dalenii (Iridaceae)

Gladiolus dalenii is another common traditional herbal medicine in Africa. The extract of Gladiolus dalenii is reported that it has a strongly anticonvulsant property in pentylenetetrazol-induced seizures by modulatory reaction on GABA_A_ receptor, and it can significantly decrease MES-induced seizures in animal models mediated through prolonging the inactivation of sodium channels [45–47]. Besides, Gladiolus dalenii also possesses a sedative property, and exerts a synergistic effect when combining medication with diazepam. There is no significant acute toxicity effect found at a dose less than 4 g/kg after oral administration in rats. The adverse effects, including hypoactivity, asthenia, and salivation, will happen at a higher dose than 4 g/kg [47].

### Traditional herbal medicines for epilepsy in America

#### United States traditional herbal medicine for epilepsy

As a common approach of complementary and alternative medications, traditional herbal medicine has a place in modern antiepileptic medical practice of United States [8]. The efficacy data of herbal medicine for epilepsy have been documented in available medicine literature. The total costs of herbal medicines in United States sum up to 690 million dollars. The herbs used for epilepsy in United States include American hellebore (*Veratrum viride*), betony (*Stachys officinalis*), Blue cohosh (*Caulophyllum thalictroides*), Kava (*Piper methysticum*), Mistletoe (*Viscum sp*), Mugwort (*Artemisia vulgaris*), Pipsissewa (*Chimaphila umbellate*), skullcap (*Scutellaria laterifolia* and *S baicalensis*), Valerian (*Valeriana officinalis*) [48–50]. Though these herbs have been used to treat epilepsy in humans, most of them have not been proven in clinical assay, even not in laboratory animal test. As the antiepileptic effects of skullcap and Valerian have been reviewed in our study above, Mistletoe and Kava also have shown positive effects in laboratory animal assay. However, the lack of clinical data places constraints on the clinical recommendation of herbal medicine [48]. Hence, the patients with epilepsy who have received herbal medication should be encouraged to report the drug response, including efficacy and adverse effects. However, further investigations should more focus on both basic and clinical data.

#### Other commonly used herbal medicines

##### Cannabis

Medical use of cannabis dates back to 2700 years before Common Era, and its use for epilepsy were reported in medieval times and late 19th century [51]. In these times, though anecdotal effective for epilepsy, there were no well-controlled trials to examine, so the use of cannabis for epilepsy remained questionable [51]. Despite presence of varying degrees defects, some recent studies, the results of which have been reviewed, have supported the antiepileptic effects of cannabis [51–57]. The antiepileptic ingredients of cannabis include Δ^9^-tetrahydrocannabinol, Δ^9^-tetrahydrocannabivarin, cannabidiol, cannabinol, and cannabidivarin, and all of them act on multiple receptor targets [51]. Δ^9^-tetrahydrocannabinol and Δ^9^-tetrahydrocannabivarin (the propyl variant of Δ^9^-tetrahydrocannabinol) are main psychoactive agents of the ingredients of cannabis, and both of them show anti-seizure effect on acute generalized seizure models, including MES- and pentylenetetrazol-induced seizures. However, not all studies agree with the results. Some studies found that Δ^9^-tetrahydrocannabinol made no positive effect on some seizure animal models, and it induced epileptic form activity [51]. Cannabidiol has been confirmed by recent studies that it has antiepileptic and anticonvulsant effects in acute models of seizure, but the detail mechanisms remain partly known [58–60]. The effects of cannabidiol on seizure may differ in types of seizure models. Some studies indicate that cannabidiol may make positive effects and well tolerated in the treatment of refractory pediatric epilepsy [55]. There are two retrospective cohorts of cannabidiol products used for children epilepsy [55, 56]. In Craig A. Press’ study, 43 patients (57%) reported improvement in seizures, 33% patients improved alertness/behavior, 11% patients improved motor skills and language, 7% patients improved sleep, and 44% patients suffered from side effects. In Shaun A’ study, 100 (85%) patients reported a decrease in seizure frequency, including 16 (14%) reporting complete seizure freedom. Side effects during cannabidiol exposure are significantly lower than those before cannabidiol exposure. Besides, improvement in sleep (53%), alertness (71%), and mood (63%) was reported. Notably, the vast majority (93%) of the respondents would like to continue taking cannabidiol products after survey completion.

Despite being well tolerated in humans, whether cannabidiol can use for patients with epilepsy is still a problem. cannabidivarin, the propyl variant of cannabidiol neglected before, has been found to show prominent anticonvulsant effects on seizures of animal models, besides, no significant adverse effects occur at anticonvulsant dose of cannabidivarin [54]. The study also investigated the combination effects of cannabidivarin and antiepileptic drugs in seizure models, and the combination significantly decreased seizure severity and the occurrence of severe seizures, and there was no significant positive or negative interaction at experiment dose. At high dose 1500 mg/day (p.o.) or 30 mg (i.v.), no significant adverse effects were seen in both acute and chronic medication [61]. Long term administration of cannabidiol may cause immunosuppression in human [51]. The anticonvulsant mechanisms of cannabis may attribute to promoting cannabinoid receptor type 1 expression and localization on different presynapses (i.e. excitatory or inhibitory), inhibiting GABAergic circuits in brain areas crucial to epileptogenesis, and inhibiting

diacylglycerol lipase α, an enzyme responsible for the production of the endocannabinoid 2-arachidonoylglycerol, to increase the number of cannabinoid receptor type 1 at the membrane [52, 53]. These results indicate that cannabidivarin has potential to treat seizures and may provoke further studies of cannabidivarin on seizures.

#### Ginseng

Ginseng is one of the most common herbal medicines used for ailments in China, Korea and America. Some studies have shown that ginsenosides play a significant role in most pharmacological effects of ginseng, including anti-inflammatory and neuroprotective effects [62–64]. Both single and chronic administration of ginseng extracts can reduce the development of neurodegeneration, inflammation and larger permeability caused by status epilepticus particularly in the hippocampus at the initial days, but cannot completely reverse the reduction of hippocampal volumes in rats for a month after status epilepticus [63]. Chronic administration of ginseng extract on rats exhibited no toxic effects, but high dose more than 150 mg/kg treatment on rats increased mortality rate after status epilepticus induced by pilocarpine [63]. It is reported that Korean red ginseng extracts also significantly increase the electrical seizure threshold in rat offspring [63]. Some studies on the neuroprotective mechanisms of ginsenosides indicate that ginsenosides inhibit NMDA-dependent and status epilepticus induced Ca^2+^ influx, and L-type Ca^2+^ channels in hippocampal neurons [62, 65, 66].

#### Pimpinella anisum

Pimpinella anisum is distributed to the eastern Mediterranean region and Asia. It is reported that Pimpinella anisum has been used for epilepsy from medieval ages in Persian medicine [67]. The recent data suggest the anticonvulsant effects of anise oil in pentylenetetrazol- and MES-induced seizures animal models [68, 69]. A study investigated the anticonvulsant effects of three different concentrations of anise oil (1 ml/kg, 2 ml/kg, and 3 ml/kg) [69]. Anise oil at all the three concentrations shows significantly anti-seizure properties in pentylenetetrazol-induced models. The anti-seizure activity may be mediated through activation of GABA_A_ receptors [70]. The main ingredients in anise oil include trans-anethole (89.1%), estragol (3.6%), linalool (1.1%), α-terpineol (0.2 %) and cis-anethole (0.2%), but all ingredients are possibly responsible for the anti-seizure effect. Further evaluations should be made on this problem.

#### Passiflora invarnata (purple passion flower)

Passiflora invarnate, a local American plant, is firstly used for epilepsy by native Americans, and its medicinal value is widely accepted by modern western medicine [71]. Some studies have shown the anticonvulsant efficacy of passiflora extracts [71, 72]. However, the active ingredients of passiflora have not been well defined. Though most available data indicate flavonoids may be the true active ingredients of passiflora [54], the latest study suggests passiflora bioactivity attributes to compound actions of several ingredients rather than signal flavonoid [72]. The mechanisms may include a combination of GABA with additional ingredients facilitating its membrane permeation, and second order positive modulation of GABA_A_ receptors by flavonoids [72, 74, 75]. Besides, the results of this study also indicate that passiflora extracts only have anticonvulsant and anxiolytic effects and no sedation and anxiogenic effects, contrasting to benzodiazepines [72]. The pharmacological effects and mechanisms of passiflora require further determination.

#### Skullcap

There are two species of skullcap plants family used as herbal medicine, and both have anticonvulsant effects. American skullcap is traditionally used on epilepsy by Native Americans and Europeans [76]. Baikal skullcap is a common herbal medicine of traditional Chinese medicine for ailments other than epilepsy, and exhibits neuroprotective and anticonvulsant properties in some recent studies [77, 78]. A latest study has demonstrated that the extracts of American skullcap show modest anticonvulsant efficacy in pentylenetetrazol-induced models, and confirmed the effective dose range (60–150 mg/kg) and the maximum effective dose (90 mg/kg) as well [78]. There are 12 constituents isolated and purified from the whole extracts of American skullcap: 10 flavonoids and 2 phenylethanoid glycosides. The existing flavonoids in both Baikal skullcap and American skullcap may be the active ingredients contributing to the anticonvulsant effects, which may result in high affinity for GABA_A_ receptor and neuroprotective effects [77–79]. Further investigations of American skullcap and Baikal skullcap may draw some light on the development of epilepsy drugs.

#### Viscum album (Loranthaceae)

Viscum album is a native plant in Europe and Northern Asian countries, and is widely used as a herbal medicine for ailments in the folk medicine [80, 81]. It is traditionally reputed against epilepsy. Viscum album is reported that it exhibits significant antiepileptic effects in MES-, isonicotinic hydrazide acid-, and pentylenetetrazol-induced seizures model [82]. These neuroprotective properties possibly result from facilitation of GABA transmission. Though there are lots of ingredients found in Viscum album, no agents have been reported responsible for the antiepileptic effect.

#### Zingiber officinale Roscoe (ginger)

Zingiber officinale Roscoe is often used as a condiment in many countries, in some of which, it also acts as a treatment for ailments; for instance, colds, arthritis, migraines, hypertension, and so on [83–86]. One recent study on the anticonvulsant effects of ginger in timed intravenous pentylenetetrazol-induced seizure mice models has shown that different doses of ginger extracts significantly increased the threshold for the myoclonic seizures and forelimb tonic extension in comparison with control groups and be irrelative to the pentylenetetrazol intravenous time, while only higher dose of ginger significantly increased the threshold for the generalized clonic seizures [87]. The study hypothesizes that the mechanisms of anticonvulsant effects of ginger may attribute to antioxidant mechanisms, oxidative stress inhibition, calcium channels blockade, inhibiting NO production and reduces iNOS in lipopolysaccharide- stimulated mouse macrophages, levating intracellular cGMP level, and inhibiting chloride ion channel in the complex of GABA_A_ receptors [87–90]. The antioxidant ingredients in ginger include gingerols, shogaols and some phenolic ketone derivatives [87]. However, the precise molecular mechanism of anticonvulsant effects of ginger need further exploration.

## DISCUSSION

As mentioned above, though some herbal medicines are clinically used for epilepsy, there is a lack of clinical data. The antiepileptic efficiency and mechanism of herbal medicine are mostly tested in animal models. Though some antiepileptic mechanisms of herbal medicines have been revealed in available literature, there are still so many issues to explore.

The antiepileptic and anticonvulsive mechanisms of most herbal medicines reviewed in our paper are further described in details and summarized in Table 1. While many herbals have multiple action targets and mechanisms, some are reported to express antiepileptic effect by single mechanism, but it does not mean the only way. The potential action targets related to antiepileptic and anticonvulsive effects contain neurotransmitter and receptor systems (such as GABA and GABA receptors, Glutamate and Glutamate receptor), ion channels (such as Calcium channel, Sodium channel and Chloride ion channel linked to GABA receptors), antioxidant effect, immune regulating, specific receptors (such as cannabinoid receptor type 1), gene expression, other active substances (such as neurotrophin-4, N-Cadherin, diacylglycerol lipase α, and CaMK IIα).

**Table 1:** The anticonvulsive and antiepileptic mechanism of herbal medicine

Herbal Medicine	Region	Cells/Animals/Humans	Seizure Model	Action Target	Mechanism & Ref.
Apocynaceae	Africa	*In vitro* model (cerebral cortex from rats)	-	GABAA receptor	Enhances GABA’s affinity to the GABAA-receptor [37–38].
Bryonia alba	Iran	*In vitro* model (cerebral cortex from rats)	-	Benzodiazepine site on the GABAA receptor	Enhances the receptor sensitivity for endogenous GABA [11]
Caesalpinia bonducella	Iran	Animal model(mice)	MES Pentylenetetrazol Picrotoxin	Chloride ion channel linked to GABA receptors	Blocks chloride ion channel linked to GABA receptors [14].
Cannabis	Asia, Europe and so on	In vivo and in vitro models	Pentylenetetrazol Pilocarpine	cannabinoid receptor type 1 GABAergic circuits Diacylglycerol lipase α	Promotes cannabinoid receptor type 1 expression and localization on different presynapses (i.e. excitatory or inhibitory) [52]. Inhibits GABAergic circuits in brain areas crucial to epileptogenesis [52, 53]. Inhibits diacylglycerol lipase α, which is the enzyme responsible for the production of the endocannabinoid 2-arachidonoylglycerol, to increase the number of cannabinoid receptor type 1 at the membrane [52].
Compound herbal medicine	China	humans	-	NMDA receptors Calcium channel	Prolong the attenuation duration of NMDA receptor channels and alter the expression of NMDA receptor subunits [36]. Reducing in the concentration of intraneuronal Ca2+ inside the hippocampal neurons [36].
Euphorbiaceae	Africa	*In vitro* model (cerebral cortex from rats)	-	GABAA receptor	Enhances GABA’s affinity to the GABAA- receptor [40].
Ficus platyphylla	Nigeria	Animal model(mice)	Pentylenetetrazol Aminophylline Picrotoxin Strychnine Isonicotinic hydrazide acid	Calcium channel NMDA receptor Glycine Free radicals GABAergic Glutamatergic receptors	Inhibition of T-type Ca^2+^ currents [41]. Blocks glutamatergic neurotrans- mission mediated by the NMDA receptor [41]. Interference with postsynaptic inhibition mediated by glycine, an important inhibitory transmitter to motor neurons and interneurons in the spinal cord [41]. Scavenging action of free radicals (such as reactive oxygen and nitrogen species) [41]. Affinity for GABAergic [42]. Affinity for glutamatergic receptors, and reduction of glutamate release [42].
Ganoderma lucidum	China	In vivo and in vitro models	-	Neurotrophin-4 N-Cadherin Epileptic hippocampus Ca^2+^ concentration CaMK IIα expression	Promotes NT-4 expression, NT-4 can promote neuron survival, alleviate neuronal injuries, inhibit neurons from apoptosis and adjust the synapses plasticity [24]. Indirectly inhibits mossy fibers sprouting and adjust the synaptic reconstructions by inhibiting the expression of N-Cadherin [24]. Inhibits calcium overload to prevent an epileptic episode induced by Mg2+ deficiency [28]. Promotes the expression of CaMK IIα to prevent the onset of epilepsy [28].
Ginseng	America, China and so on	Animal model(rats)	MES	Ca^2+^ influx and channels	Inhibits NMDA-dependent and status epilepticus induced Ca2+ influx, and L-type Ca2+ channels in hippocampal neurons [62, 65, 66].
Gladiolus dalenii	Africa	Animal model(mice)	MES Pentylenetetrazol	GABA_A_ receptor Sodium channel	Interacts with the GABAA neuro-transmission specifically through the benzodiazepine site receptor [47]. Increase of the GABA brain content [47]. Prolongs the inactivation of sodium channel [47].
Lamiaceae	Africa	*In vitro* model (cerebral cortex from rats)	-	GABA_A_ receptor	Enhances GABA’s affinity to the GABAA-receptor [40].
Lavandula officinalis	Iran	Animal model (male mice)	Pentylenetetrazol	Glutamate release GABA receptors Calcium channel	Prevents glutamate induced neurotoxicity of cerebellar granular cell culture of rat pups [18, 19]. Enhance of GABA receptors [15]. Block calcium channel [17, 18].
Lavandula stoechas	Iran	Animal model(mice)	Pentylenetetrazol	Calcium channel	Blocks calcium channel [13].
Passiflora invarnate	America and Europe	In vivo and in vitro models	Pentylenetetrazol	GABA	Passiflora extracts contain a large amount of GABA, and can induce direct GABAA currents in cornu ammonis 1 hippocampal pyramidal neurons [72, 74, 75].
Pimpinella anisum	Asia and so on	In vivo and in vitro models	Pentylenetetrazol	Na^+^-K^+^ ATPase and GABAA receptors	Enhances the activity of the Na+-K+ ATPase, which inhibits both GABAA and GABAB components of inhibitory post- synaptic potentials [70].
Salvia miltiorrhiza	China	Animal model (Zebrafish and Mouse)	Pentylenetetrazol	GABA and related Agonists	A propensity to interact with path- ways irrelevant to GABA and related agonists [10].
skullcap	America, Europe and China	Animal model (rats)	Pentylenetetrazol	GABA_A_ receptor Neuroprotective effects	High affinity to GABAA receptor [77–9]. Neuroprotective effects mediated by anti-oxidation, anti-inflamemation, and anti-excitotoxicity [77–79].
Uncaria rhynchophylla	China	Animal model(rats)	Kainic acid	Pathways in both cortex and hippocampus regions Gene expression	Exhibit anti-convulsive effects by regulating immune response and neurotrophin signaling pathway [34]. Ameliorate kainic acid-induced seizures by regulating the expression of genes involved in neuron survival and inflammation [34].
Viscum album	Asia and Europe	Animal model (mice and rats)	Pentylenetetrazol MES Isonicotinic hydrazide acid	GABA	Enhances the response to GABA, by facilitating the opening of GABA-activated chloride channels [82].
Zingiber officinale	China and India	Animal model(rats)	Pentylenetetrazol	NO and iNOS NO/cGMP Pathway Chloride ion channel in the complex of GABA_A_ receptors Calcium channel Antioxidant property	Inhibits NO production and reduces iNOS in lipopolysaccharide-stimulated mouse macrophages [87, 90]. Elevated intracellular cGMP level [87]. Inhibits Chloride ion channel in the complex of GABAA receptors0 [87]. Block calcium channel [87,89]. [87, 88]
Zizyphus jujuba	India	Animal model(rats)	Pentylenetetrazol MES	Voltage-gated Channel GABA channel Glutamate and NMDA Antioxidant effect	Prolongs Na^+^ channel inactivation [21]. Inhibits effect of GABA and reduce the excitation [21]. Inhibits glutamate-induced overexcitation, reduces synaptic release of glutamate or NMDA [20, 21]. Reverses antioxidant–oxidant imbalance by decreasing NMDA levels and increasing glutathione levels [20].

GABA and GABA receptors, which mediate antiepileptic effects of many herbal medicines, are one of the important targets well documented in the literature. GABA, as the main endogenous inhibitory neurotransmitter, can produce inhibitory postsynaptic potentials and can be a natural anticonvulsant. The effect of herbal medicine on GABA and GABA receptors is an enhancement in receptor sensitivity for endogenous GABA (Bryonia alba, Viscum album), high affinity to GABA_A_ receptor (Euphorbiaceae, Lamiaceae, Apocynaceae, and skullcap), blockade of chloride ion channel linked to GABA receptors (Caesalpinia bonducella, Zingiber officinale), direct increase of GABA brain content (Gladiolus dalenii), and other ways to effect GABA and GABA receptors. Glutamate and Glutamate receptors are another significant drug target [91]. For instance, Lavandula officinalis prevents glutamate induced neurotoxicity of cerebellar granular cell culture of rat pups, Ficus platyphylla blocks glutamatergic neurotransmission mediated by the NMDA receptor, and Zizyphus jujuba inhibits glutamate-induced over-excitation and reduces synaptic release of glutamate or NMDA. Calcium channel and Sodium channel play a significant role in epilepsy, and also act as therapeutical drug targets for epilepsy. Lavandula stoechas and Lavandula officinalis block calcium channel. Ganoderma lucidum inhibits calcium overload to prevent an epileptic episode induced by Mg^2+^ deficiency. Ficus platyphylla and Ginseng inhibit L-type Ca^2+^ channels in hippocampal neurons, and Ginseng inhibits NMDA-dependent and status epilepticus induced Ca^2+^ influx. Oxidative stress is a cause or/and result of epileptic process, and oxidative damage can lead to neurodegeneration. A recent review has suggested that antioxidants express preventive effect on epilepsy [92]. In our paper, some herbal medicines have shown antioxidative effects by different pathways. Zizyphus jujube reverses antioxidant–oxidant imbalance by decreasing in NMDA levels and increasing glutathione levels. Ficus platyphylla scavenges action of free radicals (such as reactive oxygen and nitrogen species). Zingiber officinale inhibits Nitric oxide (NO) production and reduces inducible nitric oxide synthase (iNOS)in lipopolysaccharide-stimulated mouse macrophages. Some other mechanisms mediating antiepileptic effect have been listed in Table 1.

Besides, transient receptor potential banilloid type 1 (TRPV1) [93] and gap junction blockers [94], which have been reviewed in recent studies, are potential drug targets in epilepsy therapy. TRPV1, a calcium-permeable channel, is a cause of epilepsy and expressed in the hippocampus [75]. TRPV1 can be activated by oxidative stress, resiniferatoxin, cannabinoid receptor activators. Therefore, some herbal medicines may act on TRPV1; for instance, Cannabis. This hypothesis needs further investigation. Gap junctions are aggregations of intercellular channels and lead to hypersynchromous electrical activity which cause convulsive events [76]. These molecule mechanisms can be promising approaches to investigate herbal antiepileptic effect.

The pharmacological effects of herbal medicine on epilepsy exhibited discrepancy in epilepsy models. Epilepsy is a complicated pathophysiological process, and involves many mechanisms, which determine the complexity in the effects of herbal medicine on epilepsy. Though many studies have reported the efficacy of herbal medicine on epilepsy, there is a lack of clinical trial in the literature. Besides, the adverse effects of herbal medicine hinder the clinical medication. However, some herbal medicines are clinical use in some regions, especially China. Therefore, large samples, multi-center, double-blind, randomized, controlled clinical trials are still further needed for future research. Actually, most of the herbal medicines in our review need further studies in mechanism of antiepileptic effect. The antiepileptic effects of some herbal medicine even need a scrutiny of efficacy. Meanwhile, the effective ingredients of the extract from herbal medicine should be examine in detail. Besides, a herbal medicine database should be set up in the future. It is a formidable work and need cooperation and efforts of researchers all over the world.

## SUMMARY

The herbal medicine for epilepsy therapy is a worldwide clinical practice in folk or traditional medicine and modern western medicine. Hundreds of herbal medicines have been investigated in the available literatures. It's difficult to list all herbal medicine for epilepsy in one paper, so some common herbal medicines have been reviewed in our study. The overwhelming majority of these data are based on animal experiments. As data mentioned above, though the antiepileptic effects of some of them shown in animal experiments have been well documented, none of them to our knowledge has robust evidence for clinical medication. Besides, the efficacy and toxicity of the herbal medicine should be a subject to evidence-based scrutiny. However, there is a hard way to go. All of these factors place constraints on the clinical recommendation of herbal medicine. Some methodology technique and data reviewed in this paper may inspire further study and draw some light on the development of epilepsy drugs.

## Abbreviations

GABA: gamma amino butyric acid
NMDA: N-methyl-D-aspartate
MES: maximal electroshock
BDNF: brain-derived neurotrophin factor
NO: Nitric oxide
iNOS: inducible nitric oxide synthase
TRPV1: transient receptor potential banilloid type 1.
